# Electrochemical Immunosensors for Antibiotic Detection

**DOI:** 10.3390/bios9020061

**Published:** 2019-05-01

**Authors:** Aleksandra Pollap, Jolanta Kochana

**Affiliations:** Department of Analytical Chemistry, Faculty of Chemistry, Jagiellonian University, Gronostajowa 2, 30-387 Kraków, Poland; pollap@chemia.uj.edu.pl

**Keywords:** antibiotic, immunosensor, antibody, electrochemical, immunoassay, antibacterial resistance

## Abstract

Antibiotics are an important class of drugs destined for treatment of bacterial diseases. Misuses and overuses of antibiotics observed over the last decade have led to global problems of bacterial resistance against antibiotics (ABR). One of the crucial actions taken towards limiting the spread of antibiotics and controlling this dangerous phenomenon is the sensitive and accurate determination of antibiotics residues in body fluids, food products, and animals, as well as monitoring their presence in the environment. Immunosensors, a group of biosensors, can be considered an attractive tool because of their simplicity, rapid action, low-cost analysis, and especially, the unique selectivity arising from harnessing the antigen–antibody interaction that is the basis of immunosensor functioning. Herein, we present the recent achievements in the field of electrochemical immunosensors designed to determination of antibiotics.

## 1. Introduction

**Figure 1:**
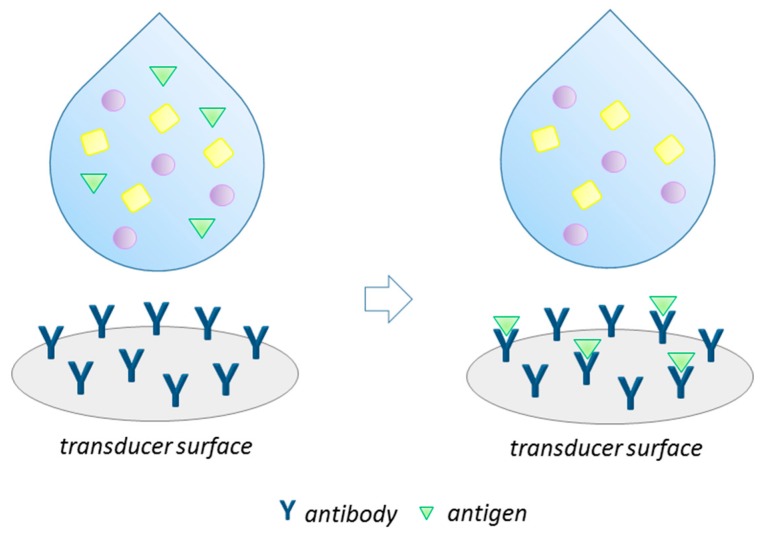
Illustration of immunosensing principle.

In recent years, a rapid development of analytical methods employing biosensors has been observed. A biosensor is a small analytical device that consists of a bioreceptor and a transducer. The role of a bioreceptor is the recognition of the target analyte, while a transducer converts the biological signal, produced by the bioreceptor and depending on the concentration of analyte molecules, into a measured signal, e.g., electrical, thermal, or optical [1]. Immunosensors constitute a class of biosensors that are based on the molecular recognition of antigens (Ag, usually the target analyte) by an antibody (Ab) on a transducer surface [2] (Figure 1). Immunodevices are attractive tools for many different types of analytes since specific antigen–antibody interactions provide the immunosensors unique selectivity and high sensitivity. They have gained wide attention due to their advantages like ease of use, simplicity, reliability, flexible application, and the amenability of integration into multifunctional analytical tools [3,4,5]. There are many reports about employing the immunosensors for use in medical diagnosis [6,7,8], food safety control [9,10,11] and environmental monitoring [12,13,14]. Immunosensors designated for determination of antibodies as target analytes have been successfully applied for medical diagnostics and early clinical diagnostics of infections, allergies, cancer, autoimmune, and cardiovascular diseases [3,5,15].

**Figure 2:**
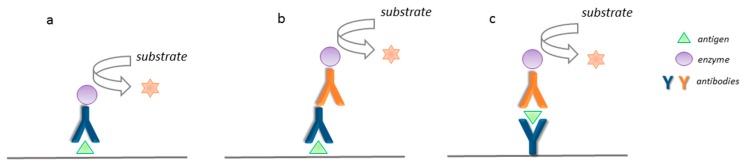
Schematic presentation of basic strategies of ELISA: (**a**) the direct ELISA, (**b**) the indirect Elisa, and (**c**) sandwich ELISA.

The idea of harnessing the immunological affinity of antibodies toward antigens was first utilized by Yalow and Bergson for human endogenous plasma insulin in 1959 [16]. Since then, a vast number of immunosensing strategies have been developed. Nowadays, the most popular immunosensors are based on the enzyme-linked immunosorbent assay (ELISA) that allows for the detection of analytes at concentration levels of 10^−12^–10^−9^ mol L^−1^ [17,18]. The simplest way to perform an ELISA test (direct ELISA) is detection of the attached solid-phase antigen of the interest on a solid-phase by the addition of the antibody labeled by an enzyme [5]. After the addition of the enzyme’s substrate, the enzymatic reaction is initiated and the signal, correlated to the antigen’s concentration, is measured. Figure 2 depicts the schematic presentation of the basic ELISA assays. In an indirect ELISA approach, in order to bind the immobilized antigen, the unconjugated antibody (primary Ab) is used, and then the secondary antibody that is covalently linked to an enzyme is introduced (see Figure 2b). The secondary antibody serves to enhance the signal of the primary antibody, which makes it more sensitive than the direct ELISA. For antigens possessing at least two binding sites (epitopes) that can interact with different types of antibodies, the sandwich format can be employed. In this approach, the antigen is caught between two antibodies that are specific for the same antigen, while one of the Ab is enzymatically labeled (see Figure 2c). Each of the ELISA realization modes presented above can be modified into a competitive format by applying either an antigen or an antibody as a competitive agent [17]. ELISAs are presently the most used and most successful techniques for immunological detection of a wide variety of antigens and the sandwich ELISA belongs to the most commonly used immunosensing formats [18].

Since the discovery of penicillin in 1929, the human race has used this powerful tool in the fight against bacterial diseases, at first it was opposed by Gram-positive pathogens [19]. Further antibiotic inventions have created the possibility of effectively controlling other bacterial infections. Therapy involved with antibiotics was one of the most important medical breakthroughs of the 20th Century [20]. Nonetheless, the availability of antibiotics, low cost of their production, and their misuse and overuse is leading to the widespread presence of antibiotics in the environment, in animals, in humans and food. As a consequence, some microorganisms have developed antibacterial resistance (ABR). At the same time, a small number of new antibacterial drugs have been discovered [19]. ABR can result in infectious diseases, easily treated with antibiotics earlier, which are becoming dangerous because they can lead to severe disability or even death. Inappropriate use of antibiotics is the reason for people developing frequent allergies to this type of medication [21]. Limiting the possibility of using a particular antibiotic on a particular patient may pose a serious threat to his or her health. ABR also results in additional medical care costs, estimated in the European Union to be at least €1.5 billion each year [21]. Thus, taking into account the above-mentioned reasons, actions should be taken to control and reduce the global problem of antibacterial resistance. Consequently, there is a great demand for monitoring and determination of antibiotics in various media such as food, beverages and environmental samples.

The growing phenomenon of ABR and the ubiquity of traces of antibiotics in the environment and in animal-derived foods as well as issues of antibiotic determination are widely discussed in literature. Different analytical methods have been proposed in detecting various kinds of samples. Among them are liquid chromatography and ultrahigh pressure liquid chromatography (UPLC), which are coupled with tandem mass spectrometry (MS/MS) [22,23] or time-of-flight mass spectroscopy (TOF-MS) [24], which are the most frequently applied analytical methods. These instrumental techniques exhibit high selectivity and sensitivity, low limit of detection and provide rewarding results with good precision. However, they require expensive equipment, skilled staff, are time-consuming, and costly sample pretreatment. A vast number of papers pertaining to sensors have been developed for electrochemical antibiotic determination [25,26,27]. Optical and electrochemical biosensors, including immunosensors [28,29] and aptasensors [30,31] as well as lateral flow assays [32,33], were also utilized for this purpose.

In this paper we have followed the recent achievements in the field of electrochemical immunosensors developed for antibiotics. In recent years several review articles have been published partially focused on electrochemical sensing of antibiotics [6,9,21,34,35,36]. Piro B. et al. described electrochemical immunosensors used in the detection of small organic molecules, including antibiotics [34]. Immunodevices—not only electrochemical ones—that employed magnetic nanoparticles in antibiotics detection were presented [21]. Biosensors, including those based on antibodies, which were developed for screening of antibiotic residue in food products of animal origin, were reported by Gaudin [9]. Felix F.S and Angnes L. presented various analytical applications of electrochemical immunosensing [6]. Alizadeh N. et al. discussed the advances and new perspectives of ultrasensitive bioaffinity electrochemical sensors, including those developed for antibiotics [35]. There are also reviews discussing the sensing of a particular class of antibiotics. Liu X. et al. described different types of sensors developed for tetracyclines [36], while Bottari F. et al. reviewed the electrochemical immunosensors that pertained to β-lactam antibiotics [37]. To the best of our knowledge there are no papers focused on electrochemical immunoassays used in the analysis of antibiotics.

The choice of the materials presented in the review was proceeded in the Scopus database using the terms “antibiotic”, “sensor”, and then “immuno”. Additionally, the database was searched for immunosensors designed to a specific group of antibiotics, e.g., “penicillins”, “tetracyclines”, etc. Among the articles found, those related to electrochemical detection were selected. Our searches of the database showed that the first report on electrochemical immunosensing of antibiotics was published in 2007 [38], and next one in 2010 [39]. A significant increase in the interest of employing these devices in the detection of antibiotics could be observed from 2012. Therefore, in the review articles that were published from 2012 to 2018 have been taken into consideration.

In order to present the subject more comprehensively, the brief overview of the classes of antibiotics has been presented and the most common electrosensing and signal amplification mechanisms have been outlined. The presented immunosensors have been classified by antibiotic class. Particular emphasis has been placed on functionalization of the electrode surface, which plays a key role in the sensor operation, and the clarification of the principles of analyte detection. The possibility of employing these sensors in clinical, environmental and food analysis was considered and also advances and future trends in their development have been discussed.

## 2. Electrochemical Sensing Mechanisms

In the development of immunosensors the same transducers used in biosensors are exploited. Among them the electrochemical, optical, magnetic and calorimetric are the most frequently used [5]. Electrochemical immunosensors can quantify the target analyte by employing amperometric, potentiometric, impedimetric or conductometric transducers. They are of particular interest because of the low costs of instrumentation and production, rapid analysis, high sensitivity and short response time, the ability of miniaturization and automatization, and they can be considered as a potential alternative to most advanced bioanalytical detection strategies [15]. Electrochemical immunodevices are becoming popular in food [40] and environmental analysis [13]. Nevertheless, most of them were designed for drug analysis and medical diagnosis [41,42,43].

**Figure 3:**
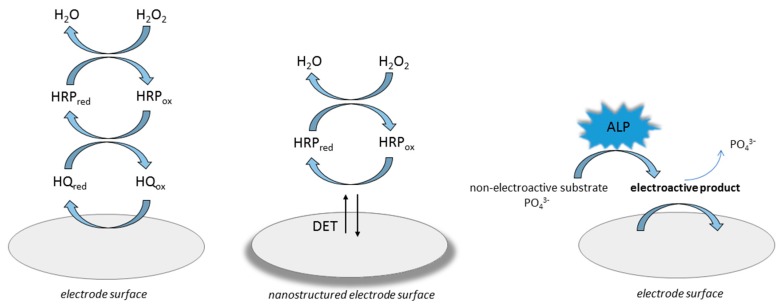
Enzymatic labeling approach. Different mechanism of electrochemical signal generation.

The most common approach for receiving an analytical signal that is generated by a transducer is employing an enzyme as a labeling component. For an enzymatically labeled electrochemical immunosensor the analytical signal that is produced can be realized in two main ways [18]. Most often the redox mediator is involved, which, in the presence of an enzyme substrate, is catalytically oxidized. In this case the registered current signal is correlated with the reduction of the oxidized mediator. Horseradish peroxidase (HRP) and hydrogen peroxide, as the enzyme substrate, are frequently utilized in this approach [3,18,44]. There are some reports regarding the direct electron transfer (DET) between the HRP redox center and the electrode surface. This approach typically required modifying the surface electrode with structures that improved the movement of electrons. DET was noticed for immunosensor labeled with HRP modified with single-walled carbon nanotube forests [45], a three-dimensional ordered macroporous magnetic Au electrode [46], and for a glassy carbon electrode modified with gold nanowires and ZnO nanorods [47]. The second strategy of enzymatic labeling is based on an application of an enzyme that catalyzes the conversion of the non-electroactive substrate to the compound that can undergo a redox reaction at the electrode surface [3,18]. The application of alkaline phosphatase (ALP) was often reported for this purpose [42,48]. Figure 3 presents the most common strategies of electrochemical signal generation using HRP or ALP as an antigen or an antibody label.

To fabricate enzyme-labeled electrochemical immunosensors that exhibit exceptionally high sensitivity, a new strategy was reported that was based on utilizing antibodies loaded with carriers that were able to support multiple enzymes [3,18]. In this approach, enzymes were conjugated to various carriers, containing, for example, metal or polymer nanoparticles, carbon nanomaterials, or magnetic beads. Due to a high label-to-carrier ratio, a dramatic signal increment was detected.

Lately, the employment of new types of labels of electrochemical immunosensors has been initiated: metal nanoparticles (NPs) and quantum dots (QDs) [18]. They constitute attractive signal tags due to their privileged merits including high surface area, biocompatibility and chemical stability. The most widely known metal nanoparticles (Au and Ag), metal sulfide (CdS, PbS, and ZnS) and semiconductor-based (ZnSe and CdTe) quantum dots were reported in the development of enzyme-free immunosensors. The determination in this mode consists of dissolution of both NPs and QDs in acidic solutions and then the detection of released metal cations by using sensitive voltammetric technics, such as stripping voltammetry [3,18]. The electrochemical immunosensors labeled with QDs [49,50] are recently much more often reported than those labeled with nanoparticles.

Currently, the signal amplification approach based on nanomaterials and their composites plays an important role in the configuration of electrochemical immunodevices. On one hand, diverse nanostructured materials exhibiting outstanding catalytic properties are intensively exploited in the construction of immunosensors in order to improve electrode conductivity and promote electron transfer [3,18]. On the other hand, they are utilized as components that enhance the construction of sensors [3]. Due to their high surface-to-volume ratio they provide a large area for the immobilization of antibodies and for more conformational freedom, favorably affecting the sensor sensitivity and lowering the limit of detection. Moreover, nanomaterials, such as NPs, carbon nanotubes, or graphene, can act as nanovehicles on the surface of which antibodies or electrochemical labels are coimmobilized, thus improving sensor performance [3]. Various nanomaterials and their nanocomposites have been proposed including gold nanoparticles [51], gold nanorods [52], metal oxide nanofiber [53], nanostructured mineral [54], carbon nano-onions [55] and microporous carbon spheres [56]. Metal–organic frameworks were also reported in the construction of immunosensors in order to encapsulate QDs and form multi-core-shell nanoparticles [50].

## 3. Antibiotics and Their General Characteristics

As mentioned before, the history of antibiotics goes back to 1929 when the first antibacterial substance–penicillin was discovered by Alexander Fleming [57]. Until recently antibiotics were referred to as substances that were naturally formed by microorganisms which are able to kill or retard the growth of other microorganisms. Nowadays, the definition of antibiotics has been extended to include antimicrobial agents coming from a synthetic or semisynthetic source [58]. We can distinguish between two types of antibiotics, bactericidal substances that are able to kill whole bacteria and bacteriostatic substances that inhibit their growth [59]. According to Chrisitan Gram’s method, based on the ability of cells to maintain the methyl violet dye after being washed, the bacteria can be divided into Gram-positive and Gram-negative, retaining or not retaining the stain, respectively [58]. The effect of antibiotics on a given type of bacteria depends on its chemical structure. The specified structural group of antibacterial substances exhibits similar efficiency, toxicity and potential side effects [59]. Due to the chemical structure of antibiotics the variety of classes could be distinguished: tetracyclines, sulfonamides, β-lactams, phenicols, quinolones, macrolides, anthracyclines, glycopeptides, aminoglycosides, and oxazolidinones [57,58,59,60]. The classification of antibiotics according to their chemical structure is shown in Table 1.

Tetracyclines are characterized by their linear four-ring skeleton with additional functional groups [61]. Their high activity against Gram-positive and Gram-negative bacteria is based on the inhibition of protein synthesis by binding to a 30S ribosomal bacteria subunit [62]. Although tetracyclines display both bacteriostatic and bactericidal activity, the bacteria are killed only using a high concentration of antibiotics [58]. Tetracyclines have found application in human treatment and in veterinary medicine used in the treatment of dairy cattle as well as animal breeding that promotes their growth [63].

Sulfonamides, also called sulfa drugs, are a popular class of antibiotics, containing the sulfonamide group in their structure and mainly exhibiting bacteriostatic properties against Gram-positive and Gram-negative bacteria [21,59]. Bacteria production is blocked by interfering with folic acid production, which plays a significant role in DNA and RNA creation [21]. They are represented by such antibiotics as sulfapyridyne, sulfadiazine, or sulfamethoxazole, and are widely used in veterinary medicine [21,57,64]. Thanks to the quickly excreting and very good solubility in urine, many antibiotics from this group are applied in treating urinary tract infections [21].

β-lactams are antibiotics based on a very reactive ring consisting of three carbon and one nitrogen atoms [59]. Their bactericidal properties result in disrupting peptidoglycan synthesis during the multiplication of bacteria. As a result the created cell walls are weak and growing cells can undergo lysis. In comparison with other groups of antibiotics, such as aminoglycosides or fluoroquinolones, their kill rate is slower and antimicrobial activity mainly depends on time, not concentration [58]. Because of their properties they are very often applied in human treatments and veterinary medicine, especially cattle [65]. As shown in Table 1, β-lactams antibiotics are divided into following groups: penicillins, cephalosporins, monobactams, and carbapenems [59]. A leading example of penicillin is penicillin G, the first discovered antibiotic [57]. The main part of this antibiotic group structure is 6-aminopenicillanic acid core [58]. Although the penicillin G has limited functionality, acting only on Gram-positive and some Gram-negative bacteria, the development of new semisynthetic antibiotics including amoxicillin or amplicillin has enabled to expand the activity spectrum of penicillin to be used against Gram-negative bacteria [59]. The term ‘cephalosporins’ refers to antibiotics characterized by a 7-aminocephalosporanic acid core. They are pharmacologically similar to penicillins and further modification expands their activity to be used against both types of bacteria [58,66]. In the structure of monobactams there is no additional ring bounded with the β-lactam ring. This group of antibiotics acts only against Gram-negative bacteria [59,67]. The last group of β-lactam antibiotics is carbapenems which exhibit a high activity spectrum used against both types of bacteria [58].

The chemical structure of chloramphenicol, which is the main representative of phenicol antibiotics and is based on the dichloroacetamide and phenyl group, demonstrates a wide spectrum of activity against Gram-positive and Gram-negative bacteria. It is usually applied in veterinary medicine for treating diseases found in both poultry and cattle [58,68].

Quinolones are based on a bicyclic core, but, since the development of new generations of quinolones, they could also include an additional ring [59]. In the structure of subsequent quinolone generations the fluorine atom usually appears at C6 position in the quinolone ring structure [59]. Because of the ability of disrupting DNA replication and transcription in bacteria, quinolone antibiotics display high antibacterial activity [59,69]. Ofloxacin, a fluoroquinolone antibiotic, is widely used in human and veterinary medicine, as well as a growth-promoting agent in animal breeding [69].

The chemical structure of another antibiotic group, macrolides is based on a 14-, 15-, or 16-membered lactone ring with sugar moieties and other substituents attached to the lactone ring. Their mechanism of action against bacteria is based on blocking the attachment of amino acids to polypeptide chains, thus preventing bacterial protein synthesis [59,70]. This antibiotic group is widely used in the treatment of bronchiectasis, rhinosinusitis, or cystic fibrosis [71].

The chemical structure of the leading representatives of anthracyclines, daunomycin, and doxorubicin, is based on a tetracycline ring that is attached to daunosamine by glycoside bond [60]. Anthracycline antibiotics are applied in the chemotherapeutic treatment of cancers such as lymphoblastic leukemia [72]. The reason of their activity against cancer is not well known but most likely results from the DNA intercalation of anthracycline antibiotic [60].

The term ‘glycopeptides’ refers to compounds consisting of a cyclic peptide formed using seven amino acids bounded to two sugars [59]. They play a significant role in the treatment of diseases caused by Gram-positive bacteria by blocking the substrate that is necessary for enzymes to take part in cell wall synthesis [73]. Vancomycyn, an exemplary glycopeptide drug, can be applied in the treatment of pneumonia, endocarditis, or meningitis [74].

Multifunctional sugars containing hydroxyl and amino groups are defined as aminoglycosides [75]. However this group of antibiotics displays a broad spectrum of activity used against Gram-positive and Gram-negative bacteria, aminoglycosides are usually applied in treating more serious diseases due to their toxic properties [58]. The bacteriostatic action mechanism is based on binding to ribosomal subunit thus resulting in the blocking of protein synthesis for bacteria [59]. Their application is very common in animal breeding for pork, chicken, and beef production [75].

The last group presented is the oxazolidinones, which is a relatively new class of antibiotics. Their main representative, linezolid, is used against major Gram-positive and some Gram-negative bacteria [76]. Because of the inhibition of bacterial ribosomal protein synthesis it is used for treating endocarditis, sepsis, and osteomyelitis [77].

## 4. Recent Reports on Electrochemical Immunosensors Designated for Antibiotic Determination

The most widely used antibiotics in human and veterinary medicine are tetracyclines and β-lactams, particularly penicillins, sulfonamides, macrolides, and fluoroquinones [21,37]. Therefore, in our review, sensors developed for the detection of drugs belonging to these classes of antibiotics have been presented and discussed first. Table 2 gives an overview of the analytical characteristics of the electrochemical immunosensors proposed for different classes of antibiotics, reported in the literature.

### 4.1. Immunosensors for Determination of Tetracycline

One of immunosensors for tetracyclines determination was proposed by Conzuelo et al. [61]. To fabricate an amperometric magneto-immunosensor, a selective antibody was immobilized on the surface of a carbon screen-printed electrode modified with magnetic beads functionalized with protein G. The immunoassay involved the competitive binding between an antibiotic and a horseradish peroxidase (HRP)-labeled tracer to an antibody. Based on the addition of H_2_O_2_ as an enzyme substrate in the presence of hydroquinone (HQ) as a redox mediator (see Figure 3), the amperometric response was recorded. Analytical characteristics of the sensor towards different tetracyclines: tetracycline (TC), oxytetracycline, chlortetracycline, and doxycycline were performed. The LOD and dynamic range for tetracycline (TC) were found to be 8.9 ng·mL^−1^ and 17.8–189.6 ng·mL^−1^, respectively. The selectivity of this proposed approach was evaluated against 6 nontarget antibiotics that were frequently present in milk and other dairy products; however no significant cross-reactivity was noticed. The usefulness of the sensor was checked by analyzing 1:1 diluted whole milk solutions spiked with tetracycline with a mean recovery of 99%, and reference material with a certified content of oxytetracycline obtaining a relative error below 4%. The developed disposable magneto-immunosensor method allowed for specific and sensitive determination of tetracyclines in milk with levels below the permitted total amount of tetracyclines.

Another immunosensor for tetracycline determination was proposed by Que et al. [63]. The developed method was based on hydrogen production catalyzed by platinum in a medium containing hydrochloric acid and potassium chloride. A new signal amplification strategy, the platinum-mediated seed growth procedure, was employed for signal amplification. The proposed method was realized by the competitive binding between tetracycline and tetracycline–bovine serum albumin conjugates labeled by platinum/graphene nanosheets with an antibody captured on a gold electrode. Due to the application of the efficient signal amplification strategy, the developed immunosensor exhibited very good analytical parameters towards tetracycline, including a wide dynamic range (0.05–100 ng·mL^−1^) and a very low detection limit, 6 pg mL^−1^, in comparison with other immunosensors reported for tetracycline determination, as presented in Table 2. To verify selectivity of the proposed immunosensor, the electrochemical response in the presence of two antibiotics from different classes (streptomycin and chloramphenicol) was examined. No significant signal change for the TC was observed in the presence of both of the tested potential interfering agents. The developed immunosensor was validated in analysis of spiked food samples including honey, milk, and peanuts, resulting in recovery rates ranging from 86 to 118%.

Liu et al. reported an immunosensor based on a gold electrode modified with magnetic nanoparticles (MNPs) using chitosan (CS) as a link [78]. An anti-tetracycline antibody was immobilized on a nanoparticles surface via a COOH–NH_2_ bond. Carboxyl-Fe_3_O_4_ MNPs played a role in signal amplification to improve immunosensor sensitivity. Determination was performed using DPV (differential pulse voltammetry). The antiTC-MNPs-CS/Au sensor showed a linear response towards TC concentration from 0.08 to 1 ng·mL^−1^ and LOD was equal to 0.0321 ng·mL^−1^. The selectivity of the developed immunosensor was checked using erythromycin, chloramphenicol, gentamicin, and penicillin belonging to four different classes of antibiotics. The biggest influence on the TC signal was noticed in the presence of erythromycin and gentamicin (ca. 14% signal decrease). However, the signal change was not significant in the presence of two other antibiotics. The accuracy of the proposed sensor was examined during the TC quantification in spiked milk samples (previously extracted with ethanol and diluted with phosphate buffer solution) receiving good recovery values (96–108%). Additionally, the analysis of milk samples showed that the detection levels obtained for TC were in good agreement with those obtained utilizing commercially available ELISA.

A new approach, based on a fully integrated bio-microelectromechanical system (Bio-MEMS) containing eight gold microelectrodes (µWEs), was proposed for the impedimetric determination of tetracycline in honey samples [79]. The determination was based on the competition of TC captured on µWes towards a polyclonal TC antibody, utilizing a mixture of a fixed concentration of an antibody and TC solutions of various concentrations. Three different methods of TC immobilization on an electrode surface were verified during the immunosensor development: functionalization with 4-aminophenylacetic acid (CMA), functionalization with CMA followed by the preconcentration of a new structure of magnetic nanoparticles (MNPs) coated with poly (pyrrole-copyrrole-2-carboxylic acid) (Py/Py-COOH/MNPs) cross-linked with an antibody, and finally, direct functionalization with Py/Py-COOH/MNPs. The last construction method obtained a highly sensitive sensor characterized by an attractive limit of detection—1.2 pg·mL^−1^—the lowest of the discussed immunodevices (see Table 2). The linear response towards antibiotic concentration was found to be between 0.0001 and 1 ng·mL^−1^. The selectivity of an immunosensor towards TC was confirmed in the presence of other representatives of cyclines: chlortetracycline, doxycycline, and oxytetracycline. The proposed sensing platform was tested in spiked honey samples with good recoveries (80–98%).

### 4.2. Immunosensors for Determination of Sulfonamides

In recent years, among the immunosensors used for the determination of antibiotics from the sulfonamides class, first of all, immunosensors for sulfapyridine determination were developed. An interesting approach in this field was developed by Conzuelo et al. [80]. Authors designed and applied biofuel cell for determination of sulfapyridine (SPY) residue in milk. An immunosensor based on a sulfapyridine antibody was immobilized on a graphite rod electrode modified with protein G employed as a cathode, while a graphite electrode with immobilized cellobiose dehydrogenase with a redox polymer was used as anode. Due to the presence of a horseradish peroxidase-labeled antibiotic analog the catalytic reduction of H_2_O_2_ in the presence of a redox mediator ((2,2′-azino-bis(3-ethyl benzothiazoline-6-sulfonic acid) diammonium salt) was possible, while the presence of a competing sulfapyridine caused the displacing of an analog captured by antibodies and blocking the reduction of H_2_O_2_. Sulfapyridine was quantified based on current density changes recorded. The received calibration plot of the measurements in a 1:1 diluted milk matrix exhibited a dynamic concentration range from 5 to 55 ng·mL^−1^, and a limit of detection equal to 2.4 ng·mL^−1^.

Conzuelo and coworkers also proposed a novel method for sulfapyridine quantification based on the scanning electrochemical microscopy (SECM) [64]. The approach was based on a similar mechanism including direct competitive binding between an antibiotic and its horseradish peroxidase-labeled analog to antibodies immobilized on a glassy carbon plate modified with protein G. Sulfapyridine determination was realized by hydroquinone oxidation catalyzed by a horseradish peroxidase in the presence of H_2_O_2_ and the reduction of generated benzoquinone. SECM quantification was realized by using a sample generator/tip collector (GC) mode by plotting the dependence of the measured reduction currents as a function of the antibiotic concentration in the spots containing several different SPY/SPY-HRP competitive mixture solutions. In comparison with the previously described immunosensor, being a part of the biofuel cell [80], the proposed method exhibited a significantly lower detection limit, 0.13 ng·mL^−1^, and a wider dynamic range (0.5–56 ng·mL^−1^). It is worth emphasizing that the analytical parameters of the proposed method were designated during the measurements in the milk matrix enriched with antibiotic. The proposed immunosensing approach constitutes an interesting alternative tool for the reliable quantification of low molecular analytes by realizing rapid SECM measurements through a line scan on spots prepared with different sample solutions.

Determination of sulfapyridine using a specific antibody labeled with cadmium sulfide nanoparticles as electrochemical nanoprobes, magnetic beads, and a graphite composite electrode was reported by Valera et al. [81]. After the immunoreaction, the nanoparticles were dissolved, and released cadmium ions were reduced generating the analytical signal. The amplitude of the peak (current) and the area under the square wave voltammetry curve (charge) were calculated in order to obtain the sensor response. Favorable LOD values were obtained—0.018 and 0.015 ng·mL^−1^—for amperometric and coulombimetric detection, respectively. It was noticed that the application of antigen biofunctionalized magnetic particles allowed the matrix effect to be reduced during the honey analysis. Analytical characteristics of the proposed immunosensor were also examined in the honey matrix (after its hydrolysis in acidic media to release the sulfonamide from the sugar conjugates). The difference in the LOD value using different kinds of detection during the honey sample analysis was also noticeable (0.011 and 0.008 ng·mL^−1^, for amperometric and coulombimetric detection, respectively).

For the determination of sulfapyridine Hassani et al. proposed a bio-microelectromechanical system (Bio-MEMS) based on gold microelectrodes modified with a new structure of magnetic nanoparticles (MNPs) coated with poly(pyrrole-co-pyrrole-2-carboxylic acid) (Py/Py-COOH) [82]. Impedimetric analyses were conducted according to the competitive detection procedure with 5-[4-(amino) phenylsulfonamide]-5-oxopentanoic acid-BSA (SA2-BSA) antigens, immobilized on the gold microelectrodes surface, towards polyclonal antibody (Ab-155). The LOD achieved a result of 0.4 pg mL^−1^; this was significantly lower in comparison to other immunosensors reported for sulfapyridine quantification (see Table 2). The linear response towards SPY was noticed to range from 0.002 to 50 ng·mL^−1^. To validate the immunosensor selectivity the signals were recorded in the presence of another sulfonamides: sulfadiazine, sulfathiazole, and sulfamerazine exhibiting no significant changes. For verification purposes an immunosensor was employed to determine sulfapyridine in spiked honey samples without any complex pretreatment receiving recovery values from 73% to 94%.

Another immunosensor was proposed for determination of sulfonamide antibiotics [83]. The sensor was prepared by the immobilization of specific antibodies on a screen-printed electrode modified with 4-aminobenzoic acid. The proposed approach was based on common competitive binding between an antibiotic and a horseradish peroxidase-labeled tracer to an antibody and recording the electrochemical response for H_2_O_2_ reduction in the presence of hydroquinone as a redox mediator. Under optimal conditions, a dynamic sulfapyridine detection range from 0.6 to 64.2 ng·mL^−1^ and LOD of 0.15 ng·mL^−1^ were received. The evaluation of the immunosensor selectivity towards two nontarget antibiotics showed good selectivity of the proposed assay. During the analysis of untreated milk samples enriched with SPY, a good mean recovery value of 103% was found. The sensor was also evaluated in spiked untreated milk samples towards six sulfonamides exhibiting low limits of detection ranging between 0.12 and 8.41 ng·mL^−1^, which are far below the limits established in EU countries for the sulfonamide residue in milk and other dairy products.

For the determination of another sulfonamide antibiotic, sulfametoxazole, Cai et al. developed an immunosensor based on specific antibody immobilized on a glassy carbon electrode modified with nanoCeO_2_–chitosan [84]. The immunoassay was realized by the direct competitive binding between an antibiotic and a horseradish peroxidase-labeled tracer with an antibody captured on an electrode surface. The reduction of H_2_O_2_ catalyzed by an unbound enzyme, in the presence of thionine as an electron mediator, generated a current signal proportional to an antibiotic concentration between 0.5 and 500 ng·mL^−1^, with a limit of detection of 0.325 ng·mL^−1^. No cross-reactivity of antibodies with other antibiotics of sulfonamide class was observed. The sensor was employed to determine sulfametoxazole in spiked milk, honey and egg samples and the results were consistent with the high-performance liquid chromatography method. Additionally, the results obtained for food samples spiked with sulfamethoxazole exhibited good recoveries (from 94.8% to 105.4%).

The last presented immunosensor applied for the determination of sulfonamides antibiotics was proposed by Zhang et al. [85]. Authors reported ultrasensitive detection of sulfonamides using silver nanoparticles decorated single-walled carbon nanohorns (Ag NPs@SWCNHs) as labels. For the immunosensor preparation antigen–bovine serum albumin conjugates were captured on a glassy carbon electrode modified with gold nanodendrites. The indirect immunoassay was realized by competition between the captured antigen and the target analyte toward the primary antibody. A secondary antibody labeled with Ag NPs@SWCNHs, in the presence of nitric acid, released Ag(I) cations from an electrode surface, generating the electrochemical signal measured using linear sweep voltammetry (LSV). Under optimal conditions, a linear range of 0.33 to 63.81 ng·mL^−1^ and LOD of 0.12 ng·mL^−1^ for sulfamethazine were found. The recovery tests in previously filtered and spiked pure water and environmental water samples from a river, a pond and a lake, were conducted showing acceptable recoveries (79–119%). The proposed strategy was also verified in real river samples with the results being in good agreement with those obtained utilizing commercially available ELISA.

### 4.3. Immunosensors for Determination of β-lactams

A significant part of electrochemical immunosensors designed for antibiotics determination concerning the quantification of penicillin G (PG). Li et al. developed an immunosensor by immobilization on a glassy carbon electrode with specific antibodies in a supported bilayer lipid membrane matrix (s-BLM) modified with gold nanoparticles [86]. The direct quantification of an antibiotic was performed based on the binding between penicillin G and its antibody, performing electrochemical impedance spectroscopy measurements in K_3_[Fe(CN)_6_]/K_4_[Fe(CN)_6_] solution. A wide linear range of 3.34 × 10^−6^ to 3.34 ng·mL^−1^ and a very low LOD value of 2.7 × 10^−7^ ng·mL^−1^ were achieved. For specificity and selectivity studies the impedance change was recorded in the presence of ampicillin (β-lactam antibiotic) and streptomycin (aminoglycoside antibiotic). The signal generated for potential interfering agents was very low reaffirming the good specificity and selectivity of the developed immunosensor. The unique analytical parameters of the proposed sensor can be the result of applying gold nanoparticles deposited through the s-BLM. For verification purposes PG was determined in spiked milk samples. Before taking measurements, the samples were centrifuged (to remove fat layer) and diluted with phosphate buffer solution. The result of the analysis of the spiked milk samples agreed with the HPLC method.

Merola with his coworkers reported another immunodevice for penicillin G determination [87]. The authors tested two different competitive immunoassays employing an antibiotic or an antibody conjugation with a HRP enzyme. The first one was based on the competition between an antibiotic and antibiotic-biotin-avidin-peroxidase conjugates to an antibody immobilized in the membrane, and the second one was involved by the competition between the added penicillin and the immobilized antibiotic to the antibody–biotin–avidin–peroxidase conjugates. For both cases, commercially available amperometric electrodes for H_2_O_2_ with an overlapped Immobilon membrane, directly covered with an antigen or an antibody, were applied. Under optimal conditions, the presented immunoassays were characterized by the same LOD value, 0.087 ng·mL^−1^, however the second approach exhibited a slightly wider linear range (0.17–1.8 × 10^4^ and 0.17–2.0 × 10^4^ ng·mL^−1^ for the first and second approach, respectively). Based on cross-reactivity tests, it was concluded that the proposed immunodevice exhibited low specificity to β-lactam antibiotics, such as dicloxacillin, ampicillin, amoxicillin, and cefotaxime, and higher selectivity towards antibiotics of another class of drugs. The sensor was employed for PG determination in previously diluted unspiked and spiked river water samples, resulting in recovery rates higher than 97%.

A few months later, the same group of scientists, reported a similar device for penicillin G determination [65]. It was also composed of an Immobilon membrane covering the amperometric H_2_O_2_ electrode. In contrast to the previous approach, for the immobilization of an antibody, the bovine serum albumin was employed and the antibody was labeled by peroxidase using a biotinylation method. The quantification was realized according to the competitive mode. The modification of electrode architecture noticeably improved the analytical parameters of the method: the wide dynamic range of 0.01 to 1 × 10^5^ ng·mL^−1^ and a low limit of detection 0.003 ng·mL^−1^ for penicillin G were found. However, the selectivity towards β-lactam antibiotics remained poor. The immunosensor functioning was verified in drugs, unspiked, and spiked samples of milk, urine, and serum, obtaining good recovery (>97%). Both proposed immunoassays were reported as highly sensitive, inexpensive and easily reproducible analytical devices.

A similar electrochemical mechanism of reduction of H_2_O_2_ was employed by Wu et al. [88]. Authors applied the HRP-labeled penicillin G antibody as an immunological part and the new methylene blue as a good electron transfer mediator, both covalently immobilized on a glassy carbon electrode. Under optimal conditions, a linear PG detection range from 1.74 to 13.91 ng·mL^−1^ and an LOD of 0.61 ng·mL^−1^ were achieved. Cross-reactivity experiments showed poor specificity of the proposed sensor towards β-lactam antibiotics and good selectivity towards antibiotics of other classes (roxithromycin and clindamycin). The satisfactory recovery rates (>96%) were obtained during the verification analysis in spiked milk samples (previously defatted and dissolved with phosphate buffer solution).

Among approaches for the determination of β-lactam antibiotics the immunosensor for ampicillin was also developed [89]. Tomassetti with his coworkers developed a new direct-flow surface plasmon resonance (SPR) immunosensor and compared its performance with a conventional electrochemical immunosensor. A commercially available amperometric electrode for H_2_O_2_ was used as a transducer; it was covered with an Immobilon membrane in which the antibody was directly immobilized. The immunoassay was realized in a competitive format between an ampicillin–biotin–avidin–peroxidase conjugated and ampicillin to be measured, both free in solution, for the anti-body captured in the membrane. Horseradish peroxidase was employed as a label of immunoconjugates. A wide linear range of 0.17 to 3.49 × 10^4^ ng·mL^−1^ and a low value of LOD, 0.087 ng·mL^−1^, were obtained. It was concluded that an amperometric immunodevice provided better analytical characteristics regarding sensitivity, linearity range and LOD, in comparison to an SPR sensor. However, during a specificity test, it was found that the SPR responded primarily to ampicillin while for the conventional approach a better response was obtained for another β-lactam antibiotic—penicillin G. Both approaches were verified in spiked bovine milk, river water, and spring surface water samples with satisfactory recoveries (>95%).

### 4.4. Immunosensors for Determination of Chloramphenicol (Phenicol Class)

One of the proposed amperometric immunosensors for chloramphenicol (CAP) determination was developed by the modification of a carbon screen-printed electrode with F_e3_O_4_–Au nanoparticles coated with conjugates of bovine serum albumin and chloramphenicol, graphene sheets and Nafion [90]. An electrochemical signal using the DPV technique was recorded in solution of K_3_[Fe(CN)_6_], and its increase with CAP concentration was noticed with a linearity between 2.0 and 200.0 ng·mL^−1^. The limit of detection was 0.82 ng·mL^−1^. The selectivity of the developed immunosensor was examined in the presence of inorganic ions (Na^+^, Zn^2+^, Ni^2+^, Co^2+^, Cu^2+^, Ca^2+^, Cl^−^, and SO_4_^2−^) nitrobenzene, para-nitrophenol, glucose, fructose, tyrosine, glutamic acid, and glycine showing no significant signal changes for chloramphenicol. The proposed disposable sensor was validated in spiked milk. For its preparation, milk samples were enriched with an antibiotic, and then trichloroacetic acid solution was added for protein precipitation. Finally, the samples were centrifuged, filtered, and diluted with phosphate buffer solution. Results obtained during the analysis of milk samples agreed with those obtained by HPLC method (the difference did not exceed 3.7%). Additionally, good recovery values were obtained (96–105.2%).

Another free-labeled immunosensor for chloramphenicol quantification was based on a screen-printed carbon electrode laminated with a layer of poly (vinyl alcohol-*co*-ethylene) (PVA-*co*-PE) nanofibrous membrane, that was covalently immobilized with an anti-chloramphenicol antibody [91]. The amperometrically monitored current of reduction of nitro groups of the captured antibiotic molecules on the antibody-modified screen-printed electrodes was an analytical signal. The developed sensor exhibited a wide linear range (0.01–10 ng·mL^−1^) and a favorable limit of detection 4.7 pg·mL^−1^. The high sensitivity of the developed immunosensor was equal to 495.1 nA ng^−1^·mL; this might be related to the enhancement of the sensing surface due to the high porosity of nanofiber membranes. The specificity and selectivity of the proposed method was tested in the presence of thiamphenicol and an antibiotic belonging to different classes: amoxicillin, gentamycin, sulphamethazine, and ciprofloxacin. It was confirmed that the developed immunosensor exhibited good both parameters. The functioning of the sensor was verified in spiked milk samples without any pretreatment, giving good recovery rates (>92%).

Tomassetti et al. investigated a catalytic “direct methanol fuel cell” (DMFC) for the amperometric determination of chloramphenicol [92]. The direct quantification of an antibiotic was performed utilizing the alcohol dehydrogenase immobilized within DMFC. For comparative purposes, a conventional amperometric immunosensor was fabricated. For this an Immobion membrane was employed, in which antibiotic molecules were immobilized. The determination was performed according to competitive protocol and ExtrAvidin^®^ peroxidase was used as a marker for the labeled antigen and for antibody complex detection. It was stated that the enzymatic fuel cell enabled significantly faster and more sensitive quantification, in comparison to the constructed immunosensor and also exhibited a slightly lower limit of detection. Comparing the constructed immunosensor to other immunosensors, based on more advanced electrode nanostructures (see Table 2), it is seen that it worked in significantly higher chloramphenicol concentration ranges (3.2 µg·mL^−1^–3.2 mg·mL^−1^) and demonstrated a much less favorable LOD value equal to 969.4 ng·mL^−1^ (3 × 10^−6^ mol·L^−1^). The selectivity of the proposed method was verified in the presence of antibiotics from different classes (penicillin G, amoxicillin, cefalotin, fosfomicin, and rifamicin). During the determination of chloramphenicol in pharmaceuticals products the accuracy of the proposed method was confirmed (relative error value was lower than 8.2%).

### 4.5. Immunosensors for Determination of Quinolones

In recent years, among the approaches for quinolone antibiotics determination, immunosensors for ofloxacin, norfloxacin, and ciprofloxacin quantification were developed. He et al. reported a promising enantioselective immunosensor for determination of R- or S-ofloxacin [93]. The immunoassay was based on a dual amplification strategy using multiwall carbon nanotube–poly(L-lysine) as a matrix to immobilize the antigen and gold nanoflowers modified with a multi-HRP-antibody to enable electrochemical determination. The immunoassay was realized by means of competitive binding between the immobilized antigen (*R*- or *S*-ofloxacin) and free OFL (*R*- or *S*-enantiomer) and primary *R*- or *S*-antibody. The secondary multi-HRP-antibody produced an analytical signal of H_2_O_2_ reduction in the presence of hydroquinone as a mediator. The immunosensor showed a specific recognition of OFL enantiomers on a linear range from 0.37 to 12.8 ng·mL^−1^ and from 0.26 to 25.6 ng·mL^−1^ for *R*- and *S*-ofloxacin, respectively, with the corresponding LOD values equal to 0.30 and 0.15 ng·mL^−1^. The performed study confirmed that the constructed enantioselective immunosensor can be used to discriminate the enantiomers of OFL by using the corresponding biocomponents, including antigens and antibodies. The evaluation of the immunosensor selectivity towards compounds structurally related to OFL, such as X-ofloxacin, pefloxacin, norfloxacin, enrofloxacin, ciprofloxacin, lomefloxacin, clinafloxacin hydrochloride, and marbofloxacin suggested acceptable selectivity of the proposed assay.

A dual signal amplification approach was also employed for OFL quantification by Zang et al. [69]. For immunosensor preparation, ofloxacin–ovalbumin conjugates were immobilized on a glassy carbon electrode modified with biocompatible polypyrrole film and gold nanoclusters. For detection purposes a multi-HRP–gold nanorod–secondary antibody was used. A proposed approach based on the common mechanism, involving the reduction of H_2_O_2_ in the presence of hydroquinone as a mediator, obtained a significantly lower LOD value—0.03 ng·mL^−1^—and a wider linear range—0.08 to 410 ng·mL^−1^—in comparison with a sensor developed by He et al. [93]. The selectivity of the proposed approach was evaluated against seven nontarget antibiotics, structurally related to OFL, and no significant cross-reactivity was noticed.

Another antibiotic belonging to quinolones is norfloxacin. For its determination Liu et al. developed an immunosensor based on the poly(amidoamine) dendrimer encapsulated gold nanoparticles (PAMAM-Au), on which anti-norfloxacin monoclonal antibodies were immobilized [40]. The HRP-labeled antigen, as the signal label, was introduced to catalyze the reaction of the substrate hydroquinone in the presence of H_2_O_2_ in the competitive reaction. The linear range and LOD of the proposed immunosensor were found to be 1 to 1 × 10^4^ ng·mL^−1^ and 0.3837 ng·mL^−1^, respectively. The specificity studies showed that only for the enoxiacin antibiotic (from other tested phenicol antibiotics: fleroxacin, mariposide, and sparfloxacin) similar current results was recorded. The occurring cross-reactivity was probably caused by a similar structure of norfloxacin and enoxiacin. For recovery studies, food samples were prepared using solid-phase extraction. The sensor was tested in different kinds of samples, namely in pork, eggs and milk, and good recovery rates (91.6–106.1%) were obtained, confirming the possibility of employing the proposed immunosensor in animal-derived food quality control.

To determine ciprofloxacin, another quinolone antibiotic, Pinacho et al. applied a magnetic graphite–epoxy composite electrode containing a magnet, which also played the role of the transducer for electrochemical detection [94]. Magnetic beads modified with antifluoroquinolone antibodies and haptens, using cyanuric chloride as a cross-linker, were used as an enzyme tracer. Two alternative competitive assays were examined and no significant difference was found in the values of the analytical parameters obtained. An amperometric signal of H_2_O_2_ reduction was proportional to the concentration of analytes between 0.063 and 8.05 ng·mL^−1^ with a detection limit of 0.017 ng·mL^−1^. Employing of magnetic beads eliminated the matrix effect, thus checking the electrochemical response of ciprofloxacin in milk matrix without any pretreatment or dilution was possible, exhibiting very low LOD value (0.009 ng·mL^−1^). The proposed immunoassay was able to detect up to seven different fluoroquinolones far below the limits established in European countries for the fluoroquinolones residue in milk samples.

### 4.6. Immunosensors for Determination of Doxorubicin (Anthracyclines Class)

For determination of antibiotics from the anthracyclines class by the immunological approach only immunosensors for doxorubicin were reported. The first immunosensor for its determination, based on an antibody immobilized on stainless steel modified with gold nanoparticles electrodeposited on a thin layer of aminopropyltriethoxy–silane, by means of electrochemical impedance spectroscopy, was reported by Rezaei et al. [95]. The Fe(CN)_6_^4−/3−^ redox probe was utilized for doxorubucin quantification. The sensor exhibited a linear correlation in two concentration ranges, from 2.5 to 30.0 and from 30.0 to 100.0 pg·mL^−1^. The detection limit of 1.7 pg·mL^−1^ was achieved. The functioning of the sensor was verified in spiked human serum. For sample preparation trichloroacetic acid was added to remove proteins and after centrifugation the supernatant was diluted in phosphate buffer solution. Results showed good recovery rates (>88%).

Rezaei and his coworkers also developed another impedance immunosensor for sensitive doxorubicin determination. It was constructed employing an antibody immobilized on gold nanoparticles placed on a gold electrode modified with thiol base sol–gel [72]. The proposed immunosensor exhibited excellent analytical characteristics. Under optimal conditions, the relative charge transfer resistance (Rct) was reported to increase with doxorubicin concentration within two linear ranges of 0.1 to 1.0 and 2.5 to 50 pg·mL^−1^, with a very low detection limit (0.09 pg·mL^−1^). During recovery studies biological samples were used. For the human serum sample preparation, trichloroacetic acid was applied to remove proteins and after centrifugation the supernatant was used without special pretreatment while the urine samples were filtered and diluted in distilled water. The satisfactory recovery rates (>95%) were obtained during the verification analysis in spiked human serum and urine samples. The developed immunosensor seems to be an attractive analytical device for the determination of doxorubicin in biological samples.

### 4.7. Immunosensor for Determination of Neomycin (Aminoglycosides Class)

A paper supported immunosensors used for the quantification of neomycin as a representative of an aminoglycoside antibiotic was proposed by Wu et al. [96]. For its construction a polyclonal antibody was immobilized on a paper strip modified with single-walled nanotubes using a simple dip-dry coating method. With the increasing antibiotic concentration a lower chronoamperometric signal was recorded due to the formation of an antibiotic-antibody conjugate decreasing the charge transfer. The sensor exhibited a low limit of detection, 0.04 ng·mL^−1^, and a relatively wide linear detection range from 0.2 to 125 ng·mL^−1^. High specificity of the proposed immunosensor was proved in the presence of gentamicin belonging to the same group of aminoglycoside antibiotics. The developed immunoassay was verified by neomycin determination in spiked milk samples (previously diluted, deproteinized and filtered) with satisfactory recoveries within the range from 93.25 to 110.47%.

### 4.8. Two-Component Immunoassays

However, the simultaneous determination of two different antibiotics using immunosensors was not very common in recent years: only two two-component immunoassays were reported. An innovative electrochemical immunodevice was proposed by Liu et al. [62]. The reported immunoassay based on an application of metal sulfide nanoclusters enabled the simultaneous determination of tetracycline and chloramphenicol on the same sensing interface. Authors modified the glassy carbon electrode with gold nanoparticles and coimmobilized on its surface the conjugates of antibiotics with bovine serum albumin. At the same time anti-tetracycline and anti-chloramphenicol antibodies were conjugated on cadmium and lead sulfide nanoclusters, respectively. Due to the competitive binding of antibiotics and immobilized haptens to antibodies entrapped on nanoclusters, and the subsequent releasing of Cd(II) and Pb(II) ions from their surface in acidic media, electrochemical detection was possible. The target analytes were discriminated due to the difference of peak potential. No cross-reactivity was revealed during the analysis performed at three TC/CAP concentration levels. The proposed approach exhibited excellent analytical characteristics. The current signals were reported to increase with both analytes concentration within a dynamic range of 0.01 to 50 ng·mL^−1^. Low limits of detection—0.0075 and 0.0054 ng·mL^−1^—were found for TC and CAP, respectively. The usefulness of the developed immunosensing strategy for the simultaneous analysis of TCs and SPYs antibiotics residues was evaluated in spiked milk and honey samples (previously centrifuged and diluted with distilled water) and good recovery rates were obtained: 88–107% and 91–119% for TC and CAP, respectively.

Simultaneous immunosensing of two different antibiotics was also realized by Conzuelo and his coworkers [97]. The immunosensor for simultaneous tetracycline and sulfapyridyne detection was based on the immobilization of antibodies on screen-printed dual carbon electrodes modified with protein-G. The immunoassay involved the application of horseradish peroxidase-labeled tracers as competitors towards antibiotics during the binding to antibodies immobilized onto a dual electrode surface. The control of antibiotic concentration was carried out measuring electrochemical signals in the presence of H_2_O_2_ as an enzyme substrate and hydroquinone as a redox mediator. The selectivity of the proposed approach was evaluated against three nontarget antibiotics (penicillin G, cefapirin, and enrofloxacin) and no significant cross-reactivity was noticed. The proposed approach showed low LODs of 0.858 and 0.097 ng·mL^−1^, and wide dynamic ranges of 2.84 to 171 and 0.48 to 113 ng·mL^−1^, for TC and SPY, respectively. The immunoassay was tested in a 1:1 diluted and spiked milk sample and milk CRM with good recovery rates (from 88% to 107% for TC and from 91% to 119% for CAP), confirming the possibility of simultaneous sensitive and selective determination of two antibiotics in milk and other dairy products.

## 5. Discussion

Almost 30 reports published in the last six years on electrochemical immunosensors designated for antibiotics detection were discussed in our review. The conducted literature survey showed that the majority of reports on immunosensors pertain to the detection of the trace amounts of particular antibiotics in animal-driven food, such as honey, bovine milk, and eggs. However, the verification of a few reported strategies was performed in pharmaceutical formulations, cow urine, human serum, and environmental water samples.

When considering the attractiveness of the immunosensors described in the review, first of all the analytical parameters they exhibit should be taken into account. The accuracy of the proposed immunoassays can be considered acceptable. For the vast majority of the sensors, accuracy was evaluated by the recovery rate obtained based on the analysis of spiked samples. A few scientists estimated this parameter employing the CRM or a reference method, such as ELISA or HPLC. The obtained values of the recovery ranges ranged from 79% to 120%. For most of the sensors, the achieved LOD values were far below the limits established in some countries for the antibiotics residues in food products. It was noticed that the enrichment of the electrode modifying layer with various nanostructures favorably influenced the analytical parameters of the sensor, especially in terms of sensor sensitivity and the LOD. The use of magnetic beads (MBs) in immunosensor construction significantly reduced the matrix effect. It also had a positive effect on the analysis time by shortening the washing steps while performing ELISA. The employment of nanosized materials with high electrical conductivity, such as metal nanoparticles, nanoclusters, nanosheets or nanofibrous membranes, caused the significant amplification of the analytical signal, and as a result, improvement of sensitivity. Due to the high surface-to-volume ratio observed in these materials, a large amount of antibodies was immobilized causing a further increase in sensitivity and lowering the limit of detection. In many cases, such modifications of enzymatically labeled sensors meant the analysis could be carried out without using a redox mediator. Similar results were achieved in dual amplified immunosensors by introducing the secondary antibody. However, not only were sensors based on such sophisticated electrode architecture reported to be suitable for the determination of antibiotics at the concentration level of pg·mL^−1^. There are papers proposing simple devices that exhibit comparable analytical characteristics. Moreover, some of the developed strategies were able to perform a sensitive analysis without any complicated sample preparation. It is worthwhile noticing that there are two papers on immunosensors designed for the quantification of two antibiotics simultaneously. The first one is based on an electrode with two different antibodies both coimmobilized on the same sensing interface, and the second employs dual screen-printed electrodes. An important aspect of immunosensor characteristics is their specificity and selectivity resulting from the antibodies used. Part of the reported sensors was based on polyclonal antibodies, exhibiting lower specificity, in comparison to monoclonal ones. As a result of insufficient specificity, non-negligible cross-reactivity towards antibiotics belonging to a particular class for a few immunosensors was revealed. Monoclonal antibodies are monospecific; nevertheless their production is more complex, difficult, and expensive.

## 6. Conclusions

Drug residues in the environment nowadays are an essential problem, as they affect human and animal health. Antibiotics traces constitute a special risk due to the spreading phenomenon of antibacterial resistance. This makes the development of fast and sensitive methods for precise and accurate antibiotics monitoring necessary in every element of the environment as well as in animal-derived food and body fluids.

Electrochemical immunosensors, one of the most popular types of biosensors, have been proposed for antibiotics quantification in various kinds of samples. They combine the unique specificity of the biorecognition element with the high sensitivity of an electrochemical transducer. Regrettably, the inventions in the field of electrochemical immunosensors designated for antibiotic determination are insufficient to implement them into routine analytical protocols.

Despite the known advantages of these devices there are some limitations and challenges in their implementation in real and routine analysis. First of all, because of the variety of antibiotics appearing in the environment, sensors exhibiting high specificity are in demand. They need to be able to detect not only drugs belonging to the same class of antibiotics, but also to quantify the particular antibiotic in the presence of another, structurally similar molecule. Therefore, research on simpler and cheaper methods of producing monoclonal antibodies, characterized by their unique specificity, is necessary. At the same time, studies on devices designed for multicomponent analysis should be undertaken. Simultaneous immunoassay of two or more antibiotics could be realized by both the immobilization of different antibodies on the same sensing interface, and employing a multi-electrode biosensing platform.

An important challenge in antibiotics immunosensing is the practical application in real complicated matrices, such as in sewage waters, waste water, soil, food, and body fluids. Sensors are needed that do not require long and complex sample preparation. The time of analysis in other parameters should be taken into account, especially in the context of environmental monitoring where the ongoing verification of the level of antibiotic contamination is crucial in risk assessment. The simplifying of the sensors construction procedure, along with improving their sensitivity, is another significant issue. Further research on utilizing nanostructured materials in immunosensor fabrication, as well as the application of the multiple labeling approach, seems to be a good direction in this regard. The low costs of easy production should allow for fast fabrication of inexpensive disposable devices and simple instrumentation could enable to perform on-site analyses in real environmental conditions. Moreover, future efforts in immunodevices development should be focused on miniaturization, such as microarray, chips, and microtiter plates.

We believe the research will be continued, and, with the progress in science and technology, they will finally become quick, cheap and effective analytical tools that will fulfill the requirements set in analytical procedures in the field of control of antibiotics contamination in various media.

## Figures and Tables

**Table 1:** Classification of antibiotics due to chemical structure [51,52,53,54,59,60,66].

Class	Chemical Structure of an Exemplary Compound	Another Examples
tetracyclines	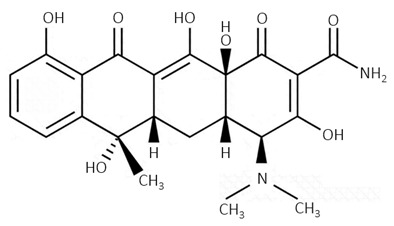 tetracycline	chlortetecycline, oxytetracycline, demeclocycline, doxycycline, lymecycline, meclocycline, methacycline, minocycline, rolitetracycline, tigecycline
sulfonamides	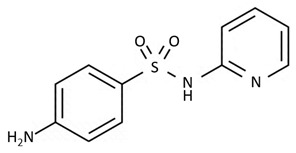 sulfapyridine	sulfadiazine, sulfamethoxazole sulfamethazine, sulfadoxine, sulfamerazine
β-lactams	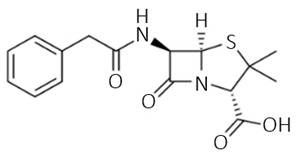 penicillin G	**penicillins:** penicillin V, dicloxacillin, methicillin, nafcillin, ampicillin, amoxicillin, carbenicilin, piperacillin, mezlocillin, ticarcillin, benzylpenicillin, cloxacillin, oxacillin, nafcillin **cephalosporins:** ceftazidime, cephazolin, cefepime monobactams: aztreonam **carbapenems: ** imipenem, meropenem, ertapenem
phenicols	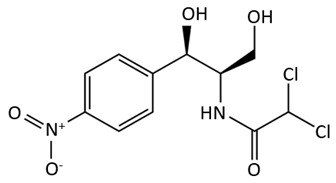 chloramphenicol	-
quinolones	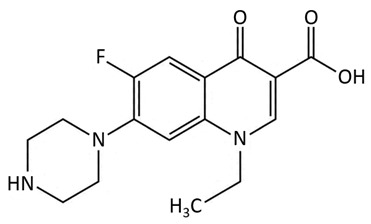 norfloxacin	cinoxacin, ofloxacin, ciproxacin, temafloxacin, sparfloxacin, nalidixic acid, enoxacin, floxacin, ciprofloxacin, levofloxacin, enrofloxacin, danofloxacin, marbofloxacin, flumequine
macrolides	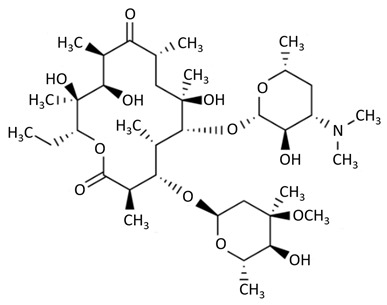 erythromycin	azithromycin, clarithromycin
anthracyclines	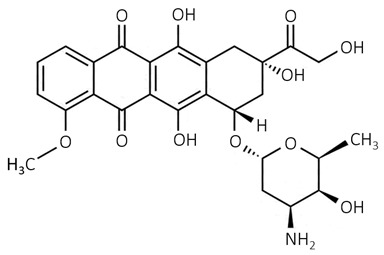 doxorubicin	-
glycopeptides	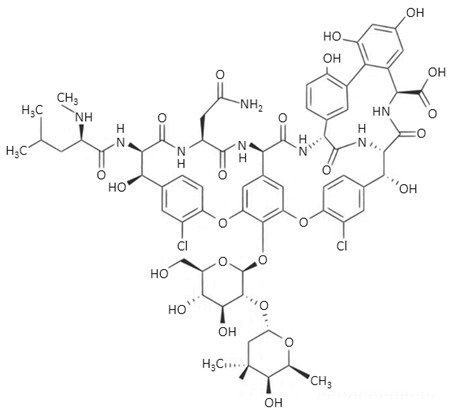 vancomycin	-
aminoglycosides	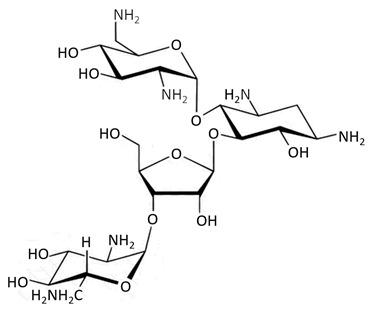 neomycin	streptomycin, gentamicin, tobramycin, amikacin, dihydrostreptomycin, kanamycin A
oxazolidinones	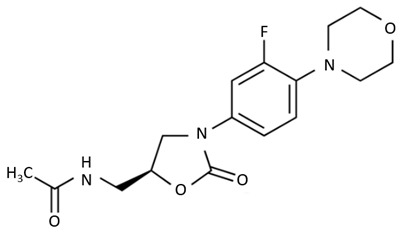 linezolid	-

**Table 2:** Characteristics of various electrochemical immunosensors developed for antibiotic quantification.

Antibiotics	Biorecognition Agent	Electrode Architecture	Detection Technique	Linear Range, ng·mL^−1^	LOD, ng·mL^−1^	Label	Selectivity	Sample	Ref.
*TETRACYCLINES*									
tetracycline oxytetracycline chlortetracycline doxycycline	anti-tetracycline polyclonal sheep antibody	antiTC-ProtG-MB/SPCE	amperometry	17.8–189.6 * 4.0–242.3 * 144.2–2001.9 * 2.6–234.9 *	8.9 1.2 66.8 0.7	HRP	+	spiked milk and CRM	[61]
tetracycline	anti-tetracycline monoclonal rabbit antibody	antiTC/GA/CS/Au	LSV	0.05–100 *	0.006	PtGNs	+	spiked honey, milk, peanut	[63]
tetracycline	anti-tetracycline monoclonal antibodies	antiTC/MNPs/CS/Au	DPV	0.08–1	0.0321	-	-	spiked milk	[78]
tetracycline	anti-tetracycline polyclonal sheep antibody	TC-Py/Py-COOH/MNPs/Au	EIS	0.0001–1	0.0012	-	+	spiked honey	[79]
*SULFONAMIDES*									
sulfapyridine	polyclonal antiserum As167	antiSPY-ProtG/GRE	amperometry	5–55 *	2.4	HRP	not available	spiked milk	[80]
sulfapyridine	polyclonal antiserum As167	antiSPY-ProtG/GCP	SECM	0.5–56 *	0.13	HRP	not available	spiked milk	[64]
sulfapyridine	antibody Ab155	GEC	SWV	-	0.015	*CdS*NP	not available	spiked honey	[81]
sulfapyridine	polyclonal antibody Ab155	SA2-BSA/Py/Py-COOH/MNPs/Au	EIS	0.002–50	0.0004	-	+	spiked honey	[82]
sulfapyridine	polyclonal antiserum As167	As167/4-ABA/SPdCE	amperometry	0.6–64.2 *	0.15	HRP	+	spiked milk	[83]
sulfamethoxazole	anti-sulfamethoxazole polyclonal antibody	antiSMX/nanoCeO_2_-CS/GCE	DPV	0.5–500	0.325	HRP	+	milk, honey, eggs	[84]
sulfamethazine	anti-sulfamethazine monoclonal antibody	SMZ-BSA/Au NDs/GCE	LSV	0.33–63.81	0.12	AgNPs	not available	environmental waters	[85]
*β-LACTAMS (PENICILLIN G)*									
penicillin G	anti-penicillin G antibody	antiP/AuNP/s-BLM/GCE	EIS	3.34 × 10^−6^–3.34	2.7 × 10^−7^	-	+	spiked milk	[86]
penicillin G	anti-penicillin monoclonal antibody (antiP)	anti-P/Immobilon membrane P/Immobilon membrane	amperometry	0.17–2.0 × 10^4^ 0.17–1.8 × 10^4^	0.087 0.087	HRP	-	spiked river, waste water	[87]
penicillin G	anti-penicillin monoclonal antibody	P-BSA/ Immobilon membrane	amperometry	0.01–1.0 × 10^5^	0.003	HRP	-	unspiked and spiked milk, urine, serum, drugs	[65]
penicillin G	anti-penicillin polyclonal antibody	anti-P-HRP/NMB/GCE	CV	1.74–13.91	0.61	HRP	-	milk	[88]
ampicillin	anti-ampicillin, monoclonal antibody	antiAMP/Immobilon membrane	amperometry	0.17–3.49 × 10^4^	0.087	HRP	-	spiked bovine milk, river water and spring surface water	[89]
*PHENICOLS*									
chloramphenicol	anti-chloramphenicol rabbit antibody	Fe_3_O_4_-Au-NPs-BSA-CAP/GS-Nafion/SPCE	DPV	2.0–200.0	0.82	-	not available	spiked milk	[90]
chloramphenicol	anti-chloramphenicol monoclonal antibody	antiCAP/PVA-*co*-PE NFM/SPCE	amperometry	0.01–10	0.0047	-	+	spiked milk	[91]
chloramphenicol	anti-chloramphenicol monoclonal	CAP/Immobion membrane	amperometry	3.2 × 10^3^–3.2 × 10^6^	969.4	ExtrAvidin^®^ peroxidase	+	pharmaceutical products	[92]
*QUINOLONES*									
*R*-ofloxacin *S*-ofloxacin	anti-*R*-ofloxacin antibody anti-*S*-ofloxacin antibody	*R*-OFL-OVA/MWCNT/PLL/GCE *S*-OFL-OVA/MWCNT/ PLL/GCE	CV	0.37–12.8 0.26–25.6	0.30 0.15	multi-HRP	+	-	[93]
ofloxacin	anti-ofloxacin antibody	OFL-OVA/Au-nanoclusters/PPy/GCE	CV	0.08–410	0.03	multi-HRP	not available	-	[69]
norfloxacin	anti-norfloxacin monoclonal antibody (antiNOR)	antiNOR/PAMAM-Au/GCE	DPV	1–1 × 10^4^	0.3837	HRP	-	spiked animal- derived food	[40]
ciprofloxacin	Ab-171 antibody	m-GEC	amperometry	0.063–8.05 *	0.017	HPR	not available	spiked milk	[94]
*ANTHRACYCLINES*									
doxorubicin	anti-doxorubicin mouse antibody	antiD-BSA/AuNP/APTES/SS	EIS	0.0025–0.03 0.03–0.1	0.0017	-	not available	spiked human serum	[95]
doxorubicin	anti-doxorubicin mouse antibody	antiD/AuNP/TB sol–gel/Au	EIS	0.0001–0.001 0.0025–0.05	9 × 10^−5^	-	not available	spiked human serum, urine	[72]
*AMINOGLYCOSIDES*									
neomycin	anti-neomycin rabbit polyclonal antibody	antiNEO/SWCNT/PSS/PS	amperometry	0.2–125	0.04	-	+	spiked milk	[96]
*TWO-COMPONENT ASSAYS*									
tetracycline chloramphenicol	anti-tetracycline monoclonal antibodies anti-chloramphenicol rabbit antibody	TC-CAP-BSA/AuNP/GCE	SWASV	0.01–50 *	0.0075 0.0054	CdS, PbS nano-clusters	not available	spiked milk, honey	[62]
tetracycline sulfapyridyne	anti-tetracycline polyclonal sheep antibody (antiTC) anti-sulfapyridyne polyclonal antiserum As167 (antiSPY)	antiSPY/antiTC/Protein G-4-ABA/SPdCE	amperometry	2.84–171 * 0.48–113 *	0.858 0.097	HRP	+	spiked milk and milk CRM	[97]

* dynamic range. antiAMP—anti-ampicillin monoclonal antibody, antiCAP—anti-chloramphenicol antibody, antiD—anti-doxorubicin mouse antibody, antiNEO—anti-neomycin antibody, antiP—anti-penicillin G antibody, antiSMX—anti-sulfametoxazole antibody, antiSPy—polyclonal antiserum As167, antiTC—anti-tetracycline antibody, 4-ABA—4-aminobenzoic acid, AMP—ampicillin APTES—3-aminopropyltriethoxysilane, Au—gold electrode, AuN—gold nanoclusters, AuNDs—Au nanodendrites, AuNP—gold nanoparticles, BSA—bovine serum albumin, CAP—chloramphenicol, CdSNP- CdS nanoparticles, CeO_2_—cerium(IV) oxide, CRM—certified reference material, CS—chitosan, CV—cyclic voltammetry, DPV—differential pulse voltammetry, EIS—electrochemical impedance spectroscopy, Fe_3_O_4_-Au-NPs—Fe_3_O_4_ and gold nanoparticles, GA—glutaraldehyde, GCE—glassy carbon electrode, GCP—glassy carbon plate, GEC—graphite composite electrode, GRE—graphite rod electrode, GS—graphene sheets, HRP—horseradish peroxidase, LSV—linear sweep voltammetry, MB—magnetic beads, MNPs—magnetic nanoparticles, m-GEC—magnetic graphite–epoxy composite, MWCNT—multi-walled carbon nanotubes, NMB—new methylene blue, OFL—ofloxacin, OVA—ovalbumin, P—penicilllin G, PLL—poly(L-lysine), PAMAM-Au—poly (amidoamine) dendrimer encapsulated gold nanoparticles, PPy—polypyrrole, ProtG—protein G, PSS—poly(sodium 4-styrenesulfonate), PS—paper strip, PtGNs—platinum/graphene nanosheets, PVA-*co*-PE NFM- poly (vinyl alcohol-*co*-ethylene) nanofibrous membrane, Py/Py-COOH—poly(pyrrole-*co*-pyrrole-2-carboxylicacid), SECM—scanning electrochemical microscopy, SMZ—sulfamethazine, SPCE—screen-printed carbon electrode, SPdCE—screen-printed dual carbon electrode, SPY—sulfapyridyne; s-BLM—supported bilayer lipid membrane, SA2-5-[4-(amino)phenylsulfonamide]-5-oxopentanoic acid, STI—soybean tripsin inhibitor, SWASV—square-wave anodic stripping voltammetry, SWCNHs—single-walled carbon nanohorns; SWV—square wave voltammetry, SS—stainless steel, TB sol–gel—thiol base sol–gel, TC—tetracycline.
